# A semi-analytical approach for the characterization of ordered 3D nanostructures using grazing-incidence X-ray fluorescence

**DOI:** 10.1107/S1600577519016345

**Published:** 2020-02-11

**Authors:** K. V. Nikolaev, V. Soltwisch, P. Hönicke, F. Scholze, J. de la Rie, S. N. Yakunin, I. A. Makhotkin, R. W. E. van de Kruijs, F. Bijkerk

**Affiliations:** a MESA+ Institute for Nanotechnology, University of Twente, The Netherlands; b Physikalisch-Technische Bundesanstalt, Berlin, Germany; c NRC Kurchatov Institute, Moscow, Russia

**Keywords:** X-ray standing wave, grazing-incidence X-ray fluorescence, periodic nano-structures

## Abstract

Following the recent demonstration of grazing-incidence X-ray fluorescence (GIXRF)-based characterization of the 3D atomic distribution of different elements and dimensional parameters of periodic nanoscale structures, this work presents a new computational scheme for the simulation of the angular-dependent fluorescence intensities from such periodic 2D and 3D nanoscale structures. The computational scheme is based on the dynamical diffraction theory in many-beam approximation, which allows a semi-analytical solution to the Sherman equation to be derived in a linear-algebraic form. The computational scheme has been used to analyze recently published GIXRF data measured on 2D Si_3_N_4_ lamellar gratings, as well as on periodically structured 3D Cr nanopillars. Both the dimensional and structural parameters of these nanostructures have been reconstructed by fitting numerical simulations to the experimental GIXRF data. Obtained results show good agreement with nominal parameters used in the manufacturing of the structures, as well as with reconstructed parameters based on the previously published finite-element-method simulations, in the case of the Si_3_N_4_ grating.

## Abstract 2

Following the recent demonstration of the sensitivity of grazing-incidence X-ray fluorescence to the lateral structure of periodic nano-patterned devices, a computational scheme for the simulation of experimental data is presented. This can be used for the element-selective analysis of 3D atomic distributions in nano-patterned structures.

## 1. Introduction   

Achievements in the field of science and technology related to the manufacturing of nanoscale devices are usually associated with the systematic decrease of the characteristic sizes of the structures within such devices. Such a decrease in characteristic sizes can lead to a strong performance dependency on minor variations in the device structure, its geometry and the elemental composition of different elements inside the structure. Prominent examples of such nanoscale device structures can be found in the microelectronics industry (Markov, 2014 ▸; Buitrago *et al.*, 2016 ▸). Understanding and improving the performance of such devices therefore requires the use of nanometrology techniques which, at best, are capable of reconstructing the geometry of the structure and the three-dimensional (3D) atomic concentration distributions of different elements. Such element-selective analysis can be performed using grazing-incidence X-ray fluorescence (GIXRF) (Soltwisch *et al.*, 2018 ▸; Andrle *et al.*, 2019 ▸). GIXRF is based on the X-ray standing wave (XSW) which is excited due to the interference between incident and reflected radiation. Its position- and angle-dependent amplitude can substantially modulate the GIXRF intensities of an element depending on its location within the nanostructure. By varying the angle of incidence and/or incident photon energy, the location of the XSW field nodes and anti-nodes can be varied inside the nanostructure. Consequently, the emission of fluorescence radiation depends on the incident angle and the incident photon energy, as well as on the spatial distribution of the fluorescent atoms.

Measurement procedures and data analysis for one-dimensional (1D) depth distributions of fluorescent atoms have been well developed (Bedzyk *et al.*, 1989 ▸; Jörg & Kazimirov, 2013 ▸) and implemented for the study of epitaxial layers (Kröger *et al.*, 2011 ▸), multilayers (Yakunin *et al.*, 2014 ▸), Langmuir–Blodgett films (Bedzyk *et al.*, 1989 ▸; Novikova *et al.*, 2003 ▸) and shallow ion implant profiles (Hönicke *et al.*, 2010 ▸), among others. However, if nanoscale devices, *e.g.* light-trapping structures in solar cells (Kröger *et al.*, 2011 ▸), field emitter arrays (Fletcher *et al.*, 2013 ▸) and nanorods (Malerba *et al.*, 2015 ▸), are to be characterized, the calculation of the XSW is more complex. The depth atomic distribution profiles of such structures can still be analyzed in the framework of the conventional 1D XSW method with use of the effective layer approximation (Kennedy *et al.*, 1999 ▸). In this approximation, the atomic concentration distribution is averaged along the lateral directions. But this approach does not take into account the diffraction on the lateral structure of the sample and is therefore only applicable in the case of randomly distributed objects. Inherently, the information about lateral distribution is lost within the effective layer approach, and the effective atomic concentration profile can never fully explain the properties of such 2D or 3D devices.

In recent works (Soltwisch *et al.*, 2018 ▸; Dialameh *et al.*, 2018 ▸), the sensitivity of GIXRF to the lateral distribution of atomic concentration in 2D and 3D structures of periodically arranged gratings and nanocolumns has been experimentally demonstrated. To achieve such sensitivity, a new experimental scheme has been employed, where measurements are carried out under different grazing-incidence and azimuthal-orientation angles. The incidence angle is typically varied within the range from zero to about 3α_c_ where the critical angle α_c_ is calculated for the bulk material. This procures formation of the standing wave in the structured layer. Variation of the azimuthal angle changes the distribution of XSW also in the lateral direction. The optical matrix method (Gibaud & Hazra, 2000 ▸) used for the analysis in conventional XSW (Yakunin *et al.*, 2014 ▸) does not allow analysis of the lateral distribution of the XSW.

This problem of GIXRF data analysis for well ordered structures has been addressed by Soltwisch *et al.* (2018 ▸), where the 2D structure of a lamellar Si_3_N_4_ grating has been analyzed. The experimentally measured GIXRF curves were analyzed by solving Maxwell’s equations by means of a finite-element method (FEM) (Pomplun *et al.*, 2007 ▸). However, applicability of FEM is limited due to its high demand in computational effort. It quickly increases with the increase of the incident photon energy, the size and the dimensionality of the structure. The FEM simulations for the experiments on the 3D Cr nanocolumns published by Dialameh *et al.* (2018 ▸) for instance are practically unrealisable.

Thus, in this study we provide an alternative approach for the calculation of the XSW field intensities within regular nanostructures by deriving semi-analytic equations based on the dynamical diffraction theory. We derive the solution of the Sherman equation (Sherman, 1955 ▸; Hönicke *et al.*, 2010 ▸) for the GIXRF intensity induced by XSW in the 3D periodic structure in linear-algebraic form. In order to test the new computational scheme, we perform numerical simulations for the same 2D lamellar grating as published by Soltwisch *et al.* (2018 ▸) and compare them with the results of FEM simulations and measurements. The semi-analytical nature of the derived equations allowed us to strongly reduce the computational effort, and to perform analysis of GIXRF also from a 3D nanostructured surface for the first time using the experimental data previously published by Dialameh *et al.* (2018 ▸).

## 2. Theory   

In Sections 2.1–2.3 we consider the theoretical background of the dynamical diffraction theory in the many-beam approximation (Mikulík & Baumbach, 1999 ▸) [in the literature also referred to as the rigorous coupled-wave analysis (Chateau & Hugonin, 1994 ▸)]. In Section 2.5 we derive the solution of the Shermann equation in linear-algebraic form, which will further allow us to calculate GIXRF intensities of 2D and 3D structures.

### 2.1. Many-beam dynamical diffraction theory   

**Figure 1:**
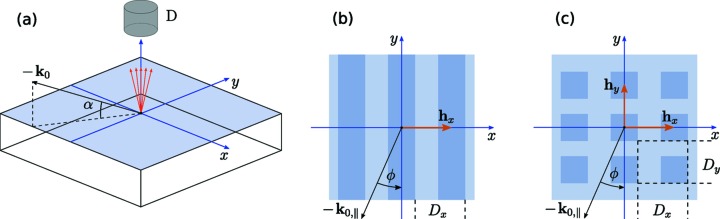
(*a*) Sketch of the experimental geometry of GIXRF. D: energy-dispersive silicon drift detector; α: angle of incidence; **k**_0_: wave vector of the incident beam. (*b*) Sketch of a 2D structure (grating: periodic in *x* direction, finite depth in *z* direction), with the azimuthal rotation angle ϕ. (*c*) Sketch of a 3D periodic structure (nanocolumns: periodic in *x* and *y* directions, finite depth in *z* direction).

The experimental geometry used by Dialameh *et al.* (2018 ▸) and Soltwisch *et al.* (2018 ▸) for the GIXRF measurements is shown in Fig. 1(*a*) ▸. An X-ray beam impinges onto a sample surface under the grazing-incidence angle α and azimuthal angle ϕ. The excited fluorescence emission is measured using an energy-dispersive silicon drift detector D. To simulate the fluorescence intensity from the sample, the near-field (NF) distribution within the nanostructure must be calculated. The problem of the NF calculation is formulated by the Helmholtz equation,
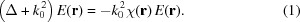
Here, for simplicity we consider the Helmholtz equation in a scalar approximation, as the effect of polarization is negligible in grazing-incidence geometry in the X-ray spectral range; 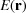 is the electric field, the sample structure is represented by the dielectric susceptibility function χ(**r**) and *k*_0_ = 2π/λ is the wavenumber of the incident beam with wavelength λ. The Helmholtz equation can be solved using the FEM, kinematical diffraction theory or dynamical diffraction theory. With FEM being computationally challenging, and kinematical theory not sufficiently precise under grazing-incidence conditions (Mikulík & Baumbach, 1999 ▸), we further consider the dynamical diffraction theory. Furthermore, to take into account the lateral structure of the sample one needs to consider the dynamical diffraction theory in the many-beam approximation (MBDDT).

In the dynamical diffraction theory, equation (1) is solved assuming that NF is represented as a Bloch wave,
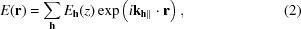
and the structure is represented as the Fourier series, 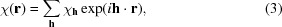
where χ_**h**_ is the Fourier component,
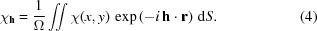
Here, integration is taken over the unit-cell area Ω for the corresponding reciprocal space vector, 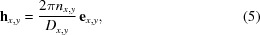
with *n*_*x*,*y*_ the order of the diffraction index and *D*_*x*, *y*_ the periods along *x* or *y* directions. The parallel component of the wavevector of the *h*th diffraction order 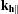 = 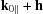 is translationally invariant along the *z* direction; *i.e.* 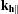 is constant across all medias in a layered system for given *h*, while the vertical component is generally different in each medium and defined with the spherical dispersion equation, 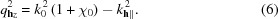
This equation is derived assuming that diffraction scattering is an elastic process: *k*_**h**_ = (1 + χ_0_)*k*_0_ and assuming translational invariance of 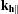 mentioned above. Finally, substituting equations (2), (3) and (6) into (1), considering a property of the Fourier components, *i.e.* 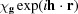 = 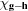, results in a system of inhomogeneous linear ordinary differential equations (ODEs) of second-order, 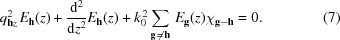
The general solution of such a system of ODEs is a linear combination of particular solutions of corresponding homogeneous ODEs, where the *n*th particular solution has the form of a standing wave with amplitudes *T*_*n*_ and *R*_*n*_. Thus, the *h*th solution of equation (7) has the form
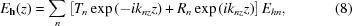
with linear combination coefficients *E*_*hn*_. Therefore, the distribution of the NF is defined with equations (2) and (8). Thus, the problem of NF calculation is reduced to finding *k*_*n*,*z*_, *E*_*hn*_, *T*_*n*_ and *R*_*n*_.

### 2.2. Characteristic equation   

In this section we discuss the calculation of *k*_*n*,*z*_ and *E*_*hn*_. Variable *k*_*n*,*z*_ has a physical meaning as the vertical component of the wavevector [see equation (8)]. It defines the phase of the standing wave in the structured layer. One can assume that *k*_*n*,*z*_ is defined with spherical dispersion *k*_*z*_ = *q*_*z*_; however, under that assumption equation (7) has no solutions. Therefore, values of *k*_*n*,*z*_ deviate from spherical dispersion. To calculate the precise value of *k*_*n*,*z*_ in the structured layer one can substitute equation (8) into (7). The result is represented as the eigenvalues–eigenvectors problem,
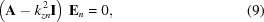
where 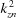 is an eigenvalue of matrix 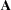 and **E**_*n*_ is an eigenvector composed of the coefficients *E*_*hn*_ from equation (8): 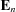 = 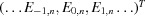; 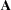 is of the form 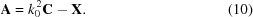
Matrix 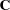 is the Toeplitz circulant matrix,
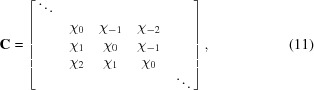
and 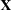 is the diagonal matrix with diagonal 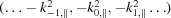. Circulant matrices have a remarkable property: with increasing circulant matrix size, their eigenvalues asymptotically approach the exact values for an infinite matrix (Gray, 2006 ▸). Therefore, one can use a finite amount of Fourier components in equation (3) to approximate the exact solution of equation (7). Consider a set of 2*N*+1 Fourier components 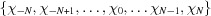. These Fourier components constitute a circulant matrix **C** of size 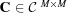, where *M* = *N* + 1. Solving the characteristic equation (9) will give *M* eigenvalue–eigenvector pairs.

### 2.3. Boundary conditions   

In this section we calculate the transmission *T*_*n*_ and reflection *R*_*n*_ amplitudes. Consider a sample as a stratified medium, consisting of layers. *T*_*n*_ and *R*_*n*_ are calculated in each layer using continuity conditions of the electric field and its first derivative. The continuity conditions (Born & Wolf, 2013 ▸) for the *j*th and (*j* + 1)th pair of layers can be written in a matrix form,
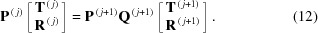
Here **T** and **R** are vectors composed of amplitudes *T*_*n*_ and *R*_*n*_, 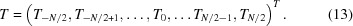
Equation (12) links amplitudes **T** ^(*j*)^, **R** ^(*j*)^ at the interface between the (*j* − 1)th and the *j*th layer, and amplitudes **T** ^(*j*+1)^, **R** ^(*j*+1)^ at the interface between the *j*th and the (*j* + 1)th layer. Matrix 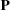 is the refraction matrix. For a structured layer it has the form
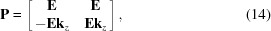
and for a homogeneous layer
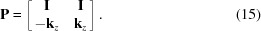
Here, the matrix 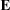 is composed of columns of eigenvectors and matrix **k**_*z*_ is a diagonal matrix filled with *k*_*z*,*n*_. Refraction matrix 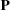 is a 2 × 2 block matrix, thus 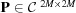. Finally 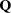 is the propagation matrix, 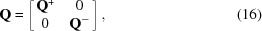
where 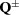 are diagonal matrices with corresponding diagonals
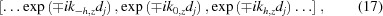
where *d*_*j*_ is the thickness of *j*th layer. Although these equations can be used to calculate **T**_*j*_ and **R**_*i*_, solving equation (12) might be problematic due to the poorly conditioned transmission matrix in the case of a sufficiently large thickness of the sample and/or in the case of a sufficiently high number of Fourier components used in the calculation.

### 2.4. Numerical stability   

**Figure 2:**
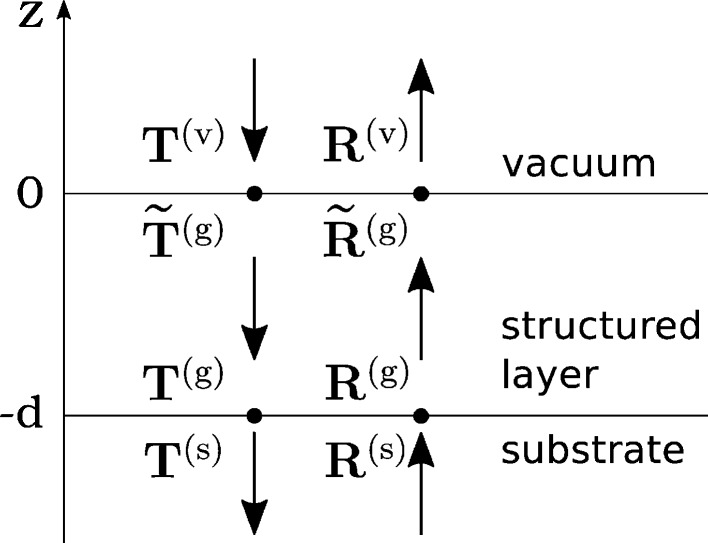
Sketch of the three-layer model. Arrows schematically depict the direction of propagation of the plane waves. Amplitudes of the plane waves are assembled into **T** and **R** vectors. 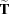 and 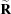 are amplitudes defined at the upper interface of the layer. **T** and **R** are defined at the bottom interface of the layer.

The problem of numerical stability in the matrix formalism of dynamical diffraction theory was considered by Stepanov *et al.* (1998 ▸). There the problem of numerical stability has been solved for the dynamical diffraction theory in a two-beam approximation (only χ_−1_, χ_0_ and χ_1_ have been taken into account). It has been solved by dividing matrices into 2 × 2 block matrices and solving equation (12) separately for each block matrix by using recurrent formula. Although the recurrent matrix equations of Stepanov *et al.* (1998 ▸) were derived for the two-beam case, they are generally applicable to the many-beam case. For brevity, we present these equations explicitly written for a three-layer system (see Fig. 2 ▸), which is relevant to the experimental data we will consider further.

The continuity conditions for such a three-layer structure (vacuum – structured layer – substrate) are represented by the system of linear equations
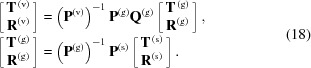
One can rewrite that system as follows,
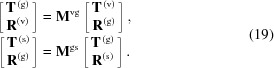
Here matrix 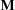 has the form of a block matrix,
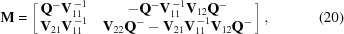
where **V**_*ij*_ is a matrix element of 2 × 2 block matrix **V** ^(vg, gs)^ = (**P** ^(v, g)^)^−1^ **P** ^(g, s)^. Note that this equation does not include 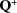 whose elements are growing exponentially with respect to the thickness of the structured layer. Hence this matrix is numerically stable. Amplitudes 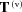 represent the incident beam, therefore 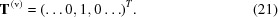
Additionally, for a sufficiently thick substrate we can assume 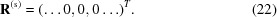
Taking into account these considerations, we derive equations for amplitudes in the structured layer,
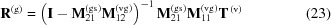
and
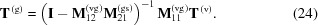
These amplitudes are calculated at the interface between the structured layer and the substrate (see Fig. 2 ▸). One can calculate amplitudes at the vacuum-structured layer interface using
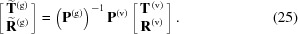
Finally, we need to rewrite equation (8),
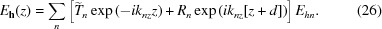
Here, both exponents decrease with respect to the depth, providing numerical stability.

### 2.5. X-ray fluorescence intensity   

The fluorescence intensity *Y* can be calculated using the Sherman equation (Sherman, 1955 ▸), adapted for GIXRF (Hönicke *et al.*, 2010 ▸),
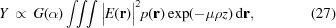
where 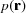 describes the density distribution of fluorescent atoms in the structure, and *G*(α) is the geometrical factor (Beckhoff, 2008 ▸; Li *et al.*, 2012 ▸; Lubeck *et al.*, 2013 ▸). The integral is taken over the area of the elementary cell. The exponential term 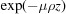 in equation (27) takes into account the self-absorption of emitted fluorescent photons. Here, μ is the absorption coefficient and ρ is the effective density of the absorbing media. The integral in equation (27) can be separated as follows [further, for brevity we do not explicitly write the multiplicative term *G*(α)],
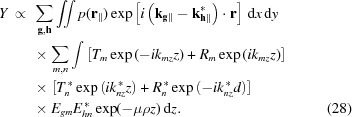
Such an integral separation imposes a restriction on the numeric density function: it must not be dependent on the *z* coordinate, *p*(**r**) ≡ *p*(**r**_α∥_); *i.e.* equation (28) can only be used in cases when fluorescent atoms are distributed homogeneously along the *z* direction. Distribution in the *xy*-plane can be arbitrary. In the case of an inhomogeneous vertical distribution, one can discretize the structure along the *z* direction as a stack of sublayers and calculate equation (28) for each sublayer.

Equation (27) was rewritten in the form of equation (28), so it can be conveniently represented in a linear algebraic language. The fluorescence intensity can be expressed as the sum of matrix elements 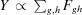 of the matrix,
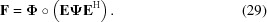
Here 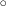 represents an element-wise (Hadamard) multiplication and H represents a Hermitian transpose. Elements of matrix 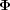 have the form
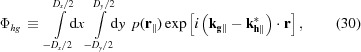
and elements of matrix 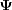 have the form
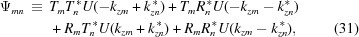
where
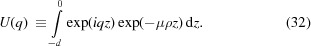
The 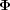 matrix takes into account the distribution of fluorescent atoms and the electric field distribution in the lateral direction and the 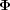 matrix takes into account photon absorption and the electric field distribution in the vertical direction. Equation (29) allows the integral in equation (27) to be calculated analytically, which is much more computationally efficient compared with the numerical integration.

## 3. Numerical simulations   

### 3.1. 2D structure: Si_3_N_4_ lamellar grating   

Here, we consider a 2D lamellar Si_3_N_4_ grating prepared using electron beam lithography. The original study with experimental data and numerical simulation of GIXRF intensity by means of FEM has been published by Soltwisch *et al.* (2018 ▸). The grating has a nominal period of *D*_*x*_ = 100 nm, the thickness of the structured layer is *d* = 90 nm and the line width is 40 nm.

The GIXRF measurements were carried out at the plane-grating monochromator (PGM) beamline (Senf *et al.*, 1998 ▸) for undulator radiation at the PTB laboratory (Beckhoff *et al.*, 2009 ▸) of the BESSY II electron storage ring.

An incident photon energy of 520 eV was used. The PGM monochromator provides an energy resolution of δ*E*/*E* < 5 × 10^−4^ in this energy range. The GIXRF intensities were obtained for the N-*K*α fluorescence emission under various incidence angles α and azimuthal sample orientation angles ϕ [see Fig. 1(*b*) ▸]; ϕ = 0° corresponds to the conical orientation (Goray *et al.*, 2018 ▸) of the sample grating. The recorded spectra from the silicon drift detector were deconvoluted using detector response functions in order to isolate the fluorescence signal from N-*K*α from other spectral contributions. Further corrections, to take into account the detection efficiency and the geometrical factor (effective solid angle), were applied [see Soltwisch *et al.* (2018 ▸) for further details].

**Figure 3:**
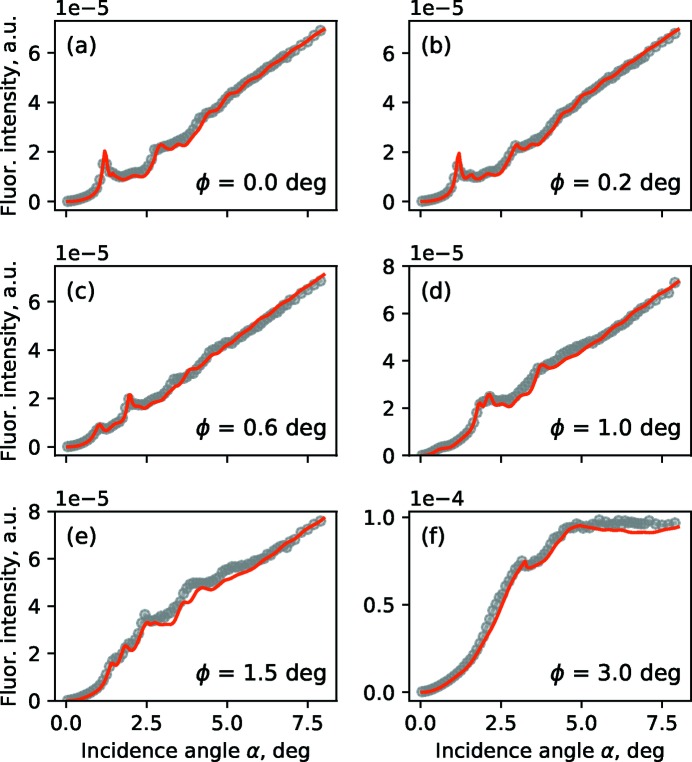
N-*K*α GIXRF intensity, measured for various azimuthal orientation angles: (*a*) ϕ = 0° – conical, (*b*) ϕ = 0.2°, (*c*) ϕ = 1° and (*d*) ϕ = 3°. Red lines: numerical simulation; gray markers: experimental values.

Best-fit simulations obtained by a sequential least-squares optimization algorithm and experimental GIXRF data are shown in Fig. 3 ▸ for various azimuthal orientation angles ϕ. For the simulation we use a simple box model in which the grating lines are treated as an array of boxes on top of the substrate [see Fig. 1(*b*) ▸]. Thus, the medium is divided into three areas: the vacuum, the structured layer in which the boxes are located, and the substrate. Within the box model, the sidewalls of the grating lines are considered to be parallel while the actual grating has a sidewall tilt angle. Based on the reconstruction of Soltwisch *et al.* (2018 ▸), this angle is not greater than β = 4°. In terms of the model it means that the Fourier transform in equation (3) is changing along the *z* axis. To compensate for that in the simulations, within the one-layer model averaged Fourier components have been used, *i.e.* 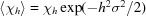, with σ defined as half the projection of the sidewall on the *x* axis: 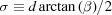. The best-fit line width (defined as the half-height width) is *D*_l_ = 39 nm and the best-fit sidewall tilt angle is β = 5°.

Another feature of the actual sample that must be considered in the simulations is the effect of oxidation of surface and line edges. It affects the actual structure such that the concentration of fluorescent N atoms at the top part and at the line edges is strongly reduced. In the one-layer model, oxidation of the surface can be effectively incorporated by changing the integration limits in equation (32), such that the integration in equation (32) is taken only over a range where fluorescent atoms are present.

The best agreement with the experimental data was obtained with an effective surface layer thickness of *d*_t_ = 3.3 nm at the top of the lines. The best fit suggests that N is not diluted at the line edges, since the reconstructed parameter of the effective edge thickness of the edges is *d*_s_ = 0 nm. We note that this value *d*_s_ is correlated with σ used in averaging of the Fourier components, and thus may not be representative. Also, note that these values only describes surface effects in terms of the absence of fluorescent N atoms, ignoring the gradual change in stoichiometry throughout the surface and the edges. It also neglects the change of optical properties of the structure due to oxidation. Best-fit parameter of the grating height, excluding the effective surface layer, is *d* = 88.7 nm. The average density of the line is 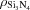 = 2.8 g cm^−3^ and the density of the substrate is ρ_Si_ = 2.22 g cm^−3^.

**Figure 4:**
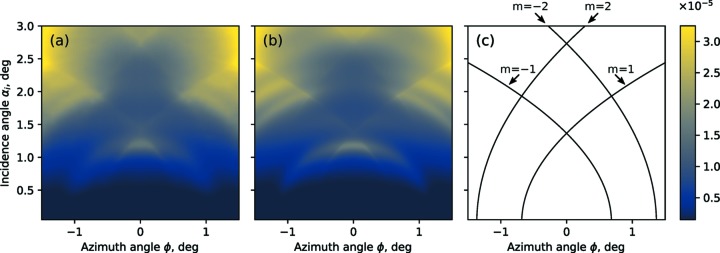
Comparison between the experimental GIXRF N-*K*α map (*a*) of the Si_3_N_4_ lamelar grating measured with the incidence photon energy *E* = 520 eV and the simulated GIXRF map (*b*) based on a best fit model. (*c*) Resonant lines in GIXRF map for Si_3_N_4_ grating structure, caused by interference between the reflected beam and the *m*th order of diffraction.

The best-fit model and experimental data are qualitatively in good agreement. Qualitative agreement is also apparent on the GIXRF intensity (α, ϕ) maps shown in Fig. 4 ▸. A full set of 48 experimental GIXRF curves taken along different azimuthal angles ϕ (from 0° to 2°) was interpolated on a (α, ϕ) grid [see Fig. 4(*a*) ▸]. The theoretical GIXRF map was calculated on the same (α, ϕ) grid using best-fit parameters from the data presented in Fig. 4 ▸.

One can note a distinctive feature on the GIXRF map – resonant lines, which are visible both on the experimental data in Fig. 4(*a*) ▸ and in the numerical simulations [see Fig. 4(*b*) ▸]. As a visual aid to notice these lines one can refer to the sketch in Fig. 4(*c*) ▸. In Fig. 4(*c*) ▸ the position of the resonant lines is marked with black contour lines.

We assume that these lines are due to the interference between the reflected beam (zeroth order of diffraction) and a diffracted beam (*m*th order of diffraction). Therefore, the resonant lines must satisfy the Laue condition, which for this geometry can be formulated as 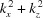 = 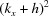. This formula geometrically corresponds to the Ewald sphere. For convenience we rewrite this equation in terms of the incidence and azimuthal angle,
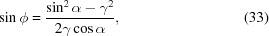
where γ = λ*m*/*D*_*x*_. The contour lines in Fig. 4(*c*) ▸ were calculated using this equation. Note that the resonant lines depend only on the lateral period of the structure *D*_*x*_ and the wavelength λ [see equation (33)] – no other geometrical parameters are involved. Due to their explicit dependence on only the period of the structure, such lines might be used in the analysis of experimental data as a reference, to determine the lateral period of the structure, without needing a full structure reconstruction through model simulations. This equation can also be used to determine the angular range of interest for the measurement as the most interesting areas to probe are around these lines.

### 3.2. 3D structure: Cr nanocolumns   

In this section we consider a periodic 3D nanocolumnar structure of Cr, manufactured using electron beam lithography (Altissimo, 2010 ▸) on top of a SiO_2_ substrate. The structure of the sample is a regular square grid of box-shaped columns [see Fig. 1(*c*) ▸] on a substrate, with 300 nm × 300 nm lateral box dimensions and a *D*_*x*_ = *D*_*y*_ = 1 µm grid. The nominal height of the nanocolumns is *d* = 25 nm.

**Figure 5:**
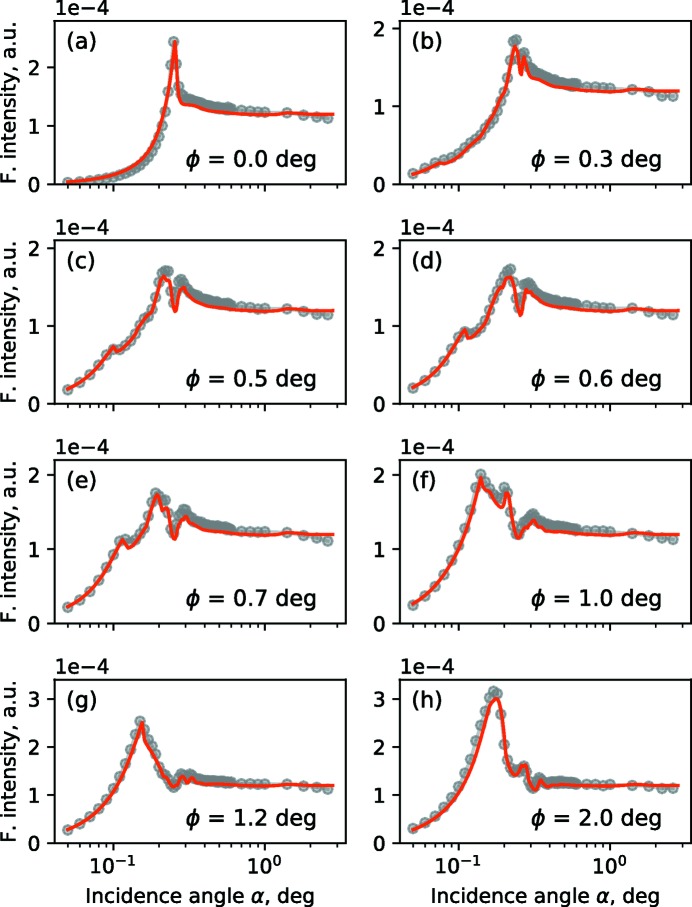
Cr-*K*α GIXRF intensity curves, measured for various azimuthal orientation angles (ϕ = 0° corresponds to the conical orientation). Red lines: numerical simulation; gray markers: experimental data.

GIXRF measurements were carried out at the four crystal monochromator (FCM) beamline (Krumrey & Ulm, 2001 ▸) in the PTB laboratory (Senf *et al.*, 1998 ▸) of the BESSY II storage ring and reported by Dialameh *et al.* (2018 ▸). The incident photon energy was set to *E* = 7 keV with an energy resolution of δ*E*/*E* < 5 × 10^−4^. Numerical simulations are carried out similarly to those in Section 3.1. The GIXRF experimental data and the best-fit obtained from dynamical diffraction theory simulations are shown in Fig. 5 ▸ for a selection of azimuthal angles.

Best-fit model parameters are: lateral period of the structure *D*_*x*_ = *D*_*y*_ = 1 µm, matching the same nominal values; lateral sizes of the nanocolumns are 300 nm × 300 nm, nanocolumn height *d* = 24 nm. The best-fit model suggests that there is no surface oxidation, *d*_t_ = 0 nm; however, the effective thickness of the sidewalls is *d*_s_ = 1.3 nm. The density of the nanocolumn material is equal to the nominal Cr density ρ_Cr_ ≃ 7.2g cm^−3^, while the substrate density is 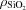 = 2.4 g cm^−3^. Considering the large lateral period *D*_*x*_ = *D*_*y*_ = 1 µm (significantly larger than that of the Si_3_N_4_ lamellar grating structure), the sidewalls tilt is negligible, therefore σ = 0 nm, *i.e.* the best-fit model for the nanocolumn structure implies perfectly parallel sidewalls 〈χ_*h*_〉 ≡ χ_*h*_. The experimental GIXRF curves in Fig. 5 ▸ are in good agreement with numerical simulations.

It is important to note that in the case of grazing-incidence geometry the GIXRF curves calculated for the 3D structure could also be approximated with the use of an effective 2D model, albeit with reduced density. This is because in the grazing-incidence geometry the momentum transfer |*k*_*y*_| ≫ |*k*_*x*_|. In other words, measurements in grazing-incidence geometry are sensitive to the lower frequencies of the Fourier transform of the structure along the *x* direction and to the higher frequencies along the *y* direction, while the spacing between nodes in reciprocal space along the *k*_*x*_ and *k*_*y*_ directions are identical due to the symmetry *D*_*x*_ = *D*_*y*_ of the periodic structure.

**Figure 6:**
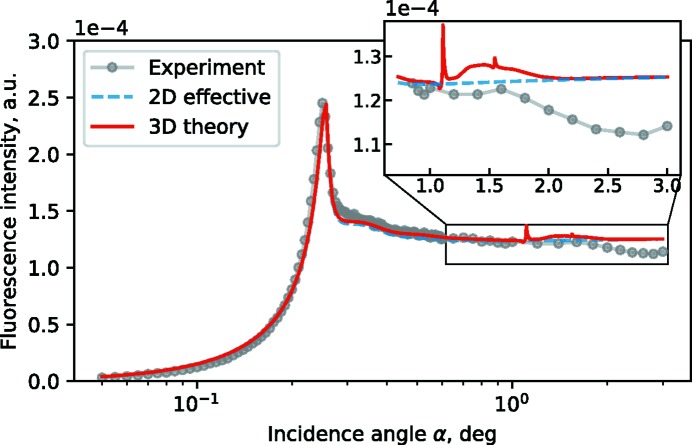
Comparison of effective 2D and genuine 3D simulations of GIXRF Cr-*K*α curve for 3D Cr nanocolumns structure in conical geometry (ϕ = 0°).

Thus, GIXRF curves of Cr nanocolumns can be effectively represented in a first approach as a lamellar Cr grating with reduced density equal to the averaged density of the actual 3D structure. However, a direct comparison between 3D and 2D simulations (Fig. 6 ▸) reveals some differences. For higher incident angles above the critical angle of total external reflection, the 2D model (dashed blue line in Fig. 6 ▸) yields a monotonous angular dependence, while the experimental GIXRF curve clearly exhibits oscillatory behaviour in that angular range, with a maximum at 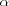 ≃ 1.5°. In Fig. 6 ▸, curves are shown only for ϕ = 0°, but this oscillation in the range of higher incidence angles α is present in all experimental curves measured at different azimuthal orientations of the sample (see Fig. 5 ▸). We attribute this oscillation to interference due to the periodicity of the structure along the *y* direction, which becomes more important at higher incident angles since the value of |*k*_*y*_| decreases with increasing incidence angle α and the measurement becomes more sensitive to the lower frequencies of the Fourier transform along the *y* direction. Such interference mode is not taken into account in the 2D simulations. Additionally, the 3D simulations show resonant peaks at α ≃ 1.15° and α ≃ 1.54° which are not resolved in experimental data. To observe these peaks, measurements with step sizes of δα = 0.01° should be resolved, which is experimentally feasible, as the resolution limit of modern synchrotron sample stage equipment is on the level of 0.001°.

## 4. Discussion   

In Table 1 we compare the structure parameters of the 2D lamellar Si_3_N_4_ grating, as reconstructed using the MBDDT simulations described in Section 2.5, with the nominal parameters used in fabrication of the grating. The results of of the MBDDT reconstruction are in good agreement with the nominal values.

**Figure 7:**
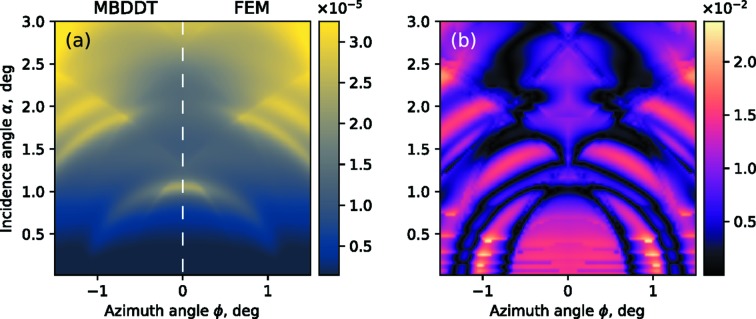
(*a*) Comparison of GIXRF maps as simulated by MBDDT (left-hand side) and FEM (right-hand side) approaches. (*b*) Relative discrepancy.

To further validate the computational scheme described in Section 2.5, FEM simulations have also been performed. The FEM simulations were performed using the *JCMwave* software (Pomplun *et al.*, 2007 ▸) for a box model based on the best-fit parameters in Table 1 ▸. *JCMwave* is a rigorous Maxwell solver, which enables field simulations in structures of arbitrary shape. For the calculation of the finite-element solution, the computational domain is meshed into patches where a number of polynomial ansatz functions are defined. The finite-element side constraint of 4 nm and a polynomial degree of 4 have been used in the simulations and the GIXRF fluorescence intensities were calculated from electric fields as described by Soltwisch *et al.* (2018 ▸). A direct comparison of the MBDDT and the FEM simulations is shown in Fig. 7 ▸. The GIXRF maps are symmetrical with respect to an axis at ϕ = 0°. In Fig. 7(*a*) ▸ the map on the left is thus showing the MBDDT result, whereas that on the right shows the FEM result. Both simulation results are visually identical. In addition, the relative discrepancy is shown in Fig. 7(*b*) ▸. Here, the relative discrepancy is defined as
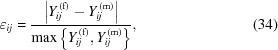
where 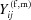 are the GIXRF intensities calculated in each (α_*i*_, ϕ_*j*_) point using the FEM and MBDDT methods, respectively.

The absolute maximum of the relative discrepancy is 2.4% and the discrepancies are generally higher for the low incidence angles. It should be noted that the precision of the FEM calculation in this angular range may be limited due to the exponential decay of the evanescent waves. In general, the relative discrepancy is on a level of 1% for 80% of the points, proving the validity of the MBDDT approach for such GIXRF simulations.

An unambiguous comparison of the computational efficiency of FEM and MBDDT cannot be performed directly, since it depends on many factors such as photon energy, period of structure, numerical accuracy, and dimensionality of the problem. For the particular simulation shown in Fig. 7 ▸, the calculation time for one point of the GIXRF map on one CPU is on the level of 10 s for FEM and 0.01 s for MBDDT. Both MBDDT and FEM computations were performed on an NUMA computer with 160 CPUs [Intel(R) Xeon(R) CPU E7-4870 v2 @ 2.30 GHz]. Thus, a calculation of the GIXRF map of 200 × 100 size on this setup takes approximately 20 min and 2 s for FEM and MBDDT, respectively.

In Table 2 ▸ we compare the MBDDT-derived structure parameters of the Cr nanocolumns with their nominal parameters. A good agreement is obtained, especially since for the current case only a simple box model is used for the numerical simulations to describe the distribution of fluorescent atoms in the structure.

With the MBDDT approach, it is also possible to take into account a structure with tilted sidewalls and surface oxidation. The model of the structure would needed to be discretized along the *z* direction, *e.g.* according to Pisarenco *et al.* (2016 ▸). The sample can be approximated as a stack of homogeneous and/or structured layers, where each layer can have arbitrary structure parameters with the exception of the period, which must be maintained throughout the whole stack.

The main benefit in applications of the 3D XSW technique to the characterization of nanostructures is its sensitivity to the spatial distribution of the fluorescent atoms within the structure. Although the examples considered in this work exhibited a homogeneous lateral distribution of N and Cr within the grating line and nanocolumn, we can still demonstrate this sensitivity by performing simple calculations.

**Figure 8:**
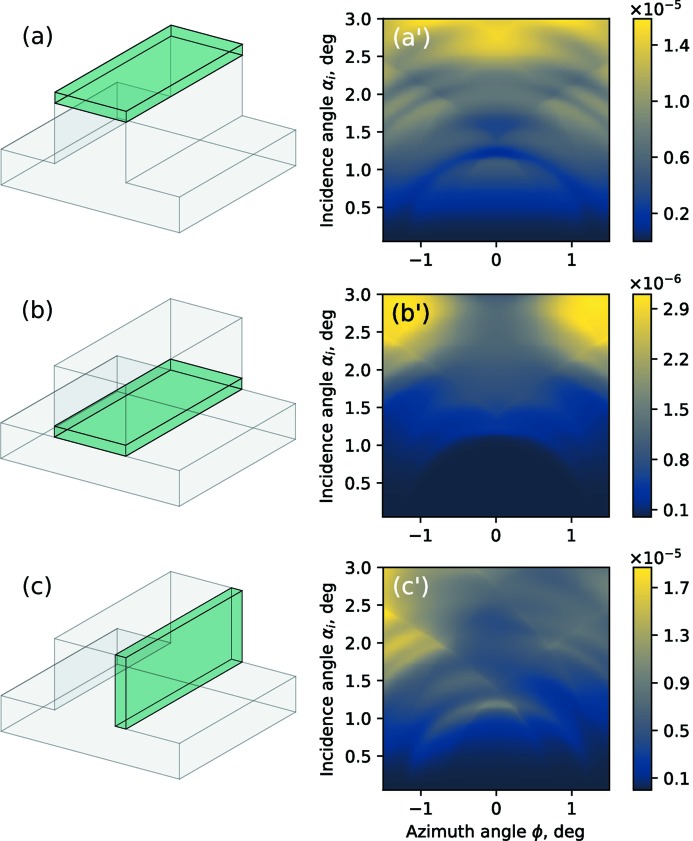
Simulation of GIXRF maps for inhomogeneous distribution of fluorescent atoms within the lamellar grating structure. From (*a*) to (*c*): sketch of the structure; the green box depicts the localization of the dopant atoms. From (*a*′) to (*c*′): corresponding GIXRF maps.

We use the same model for the Si_3_N_4_ lamellar grating as already shown earlier, but now we assume to have dopant atoms localized in a confined volume within the structure as shown in Figs. 8(*a*)–8(*c*) ▸ instead of being homogeneously distributed, as assumed in Section 3.1. The specific localization of the dopant atoms is depicted by green boxes and the resulting simulated GIXRF maps for the calculated fluorescence signal of the dopant atoms are shown. It can be observed that the corresponding GIXRF maps are highly sensitive to this variation. For an asymmetric distribution of fluorescent atoms, Fig. 8(*c*) ▸, an asymmetry is also observed in the GIXRF maps. One may exploit such asymmetry, *e.g.* to distinguish chemical compositions of the left and right sidewall of the grating line. This may be useful in, for example, the characterization of gratings fabricated with multi-patterning (Weber *et al.*, 2012 ▸) techniques.

## 5. Conclusions   

A new computational scheme based on the dynamical diffraction theory has been developed and applied for the analysis of GIXRF experiments on 2D and 3D periodic nanostructures. It is capable of simulating GIXRF data from structures with specific element distributions both in-plane as well as in-depth. The computational scheme has been validated with a Maxwell solver based on the finite-element method and benchmarked on GIXRF experimental data obtained from Si_3_N_4_ 2D lamellar gratings and Cr 3D nanocolumns. The reconstructed geometrical parameters of the lamellar grating derived from the elemental distribution are in good agreement with nominal values, as well as with parameters obtained from a previous study performed using a finite-element method. Furthermore, the parameters of the elemental distribution in the Cr 3D nanocolumns were reconstructed for the first time. A reconstruction of the geometrical parameters of this structure by means of FEM is practically impossible due to the required higher excitation photon energy, the larger period of the structures (and thus larger computational cell) and the 3D dimensionality of the sample. The obtained results of this reconstruction are in good agreement with the nominal parameters. Finally, we conclude that the MBDDT computational scheme can be used in conjunction with the GIXRF experimental technique as a powerful tool in element-selective nanometrology for 2D and 3D periodic structures.

## Figures and Tables

**Table 1:** Comparison of the 2D structure parameters of the Si_3_N_4_ lamellar grating as reconstructed by MBDDT with nominal parameters

	Nominal	Simulation
Period *D*_*x*_ (nm)	100	100
Line height *d* (nm)	87	88.7
Line width (nm)	40	39
Effective surface thickness *d*_t_ (nm)	–	3.3
Effective edge thickness *d*_s_ (nm)	–	0
Sidewalls tilt (°)	–	4
Line density 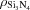 (g cm^−3^)	3.2	2.8
Substrate density ρ_Si_ (g cm^−3^)	2.33	2.22

**Table 2:** Comparison of the 3D structure parameters of the Cr nanocolumns as reconstructed by MBDDT with nominal parameters

	Nominal	Simulation
Period *D*_*x*, *y*_ (µm)	1	1
Column height *d* (nm)	25	24
Column width (nm)	300	300
Effective surface thickness *d*_t_ (nm)	–	0
Effective edge thickness *d*_s_ (nm)	–	1.3
Column density ρ_Cr_ (g cm^−3^)	7.19	7.2
Substrate density 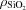 (g cm^−3^)	2.65	2.4
